# Supplementary material for: Venomix: a simple bioinformatic pipeline for identifying and characterizing toxin gene candidates from transcriptomic data
Source: PeerJ. 2018 Jul 31;6:e5361. doi: 10.7717/peerj.5361 (PMC6074769; doi:10.7717/peerj.5361)
Supplement: Supplemental Information 3 [file peerj-06-5361-s003.gz › FinalOutput_E-20/Disintegrin_EMF10B_17/finaltree.pdf]

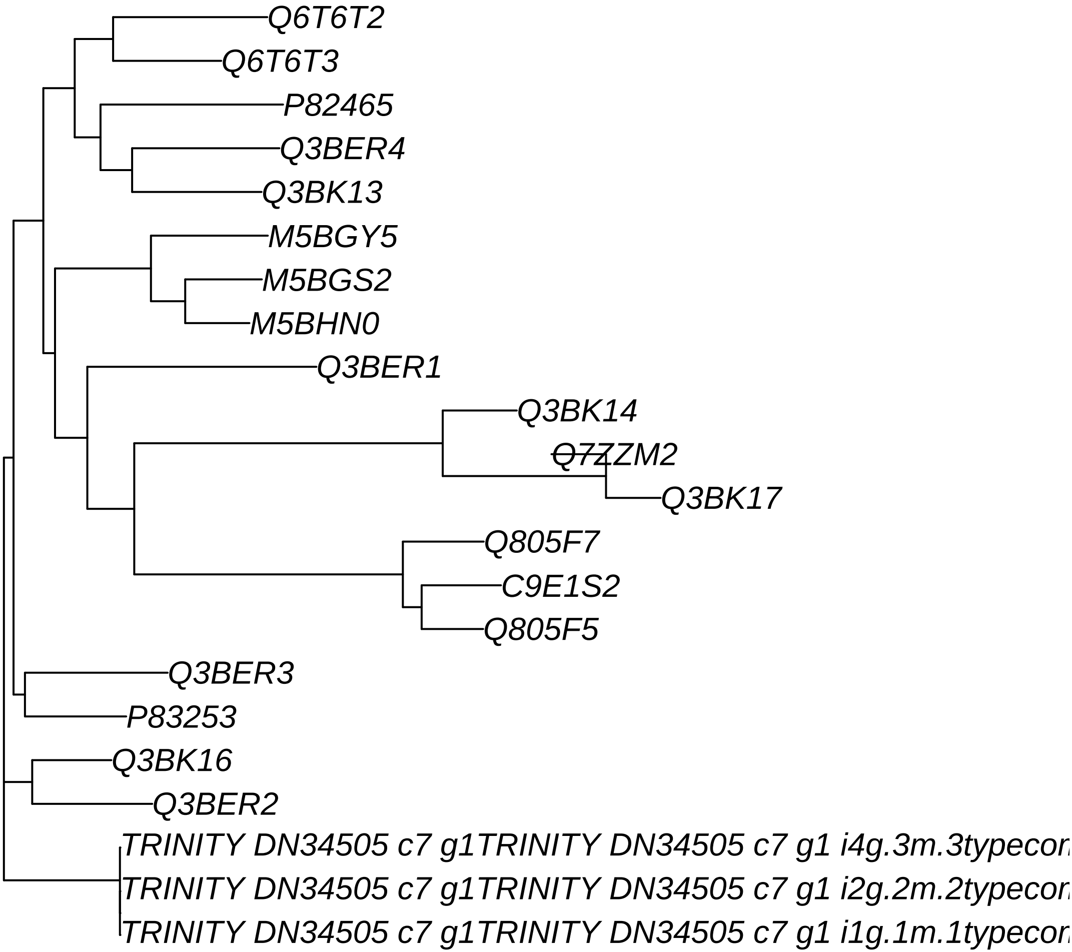

Q6T6T2
Q6T6T3
P82465
Q3BER4
Q3BK13
M5BGY5
M5BGS2
M5BHN0
Q3BER1
Q3BK14
Q7ZZM2
Q3BK17
Q805F7
C9E1S2
Q805F5
Q3BER3
P83253
Q3BK16
Q3BER2
TRINITY DN34505 c7 g1TRINITY DN34505 c7 g1 i4g.3m.3typecor
TRINITY DN34505 c7 g1TRINITY DN34505 c7 g1 i2g.2m.2typecor
TRINITY DN34505 c7 g1TRINITY DN34505 c7 g1 i1g.1m.1typecor
